# Adverse social determinants of health elevate uncontrolled hypertension risk: a cardio-oncology prospective cohort study

**DOI:** 10.1093/jncics/pkae064

**Published:** 2024-08-08

**Authors:** Priyanshu Nain, Nickolas Stabellini, Omar M Makram, Johnathan Rast, Sandeep Yerraguntla, Gaurav Gopu, Aditya Bhave, Lakshya Seth, Vraj Patel, Stephanie Jiang, Sarah Malik, Ahmed Shetewi, Alberto J Montero, Jennifer Cullen, Neeraj Agarwal, Xiaoling Wang, Bonnie Ky, Lauren A Baldassarre, Neal L Weintraub, Ryan A Harris, Avirup Guha

**Affiliations:** Department of Medicine, Division of Cardiology, and Vascular Biology Center, Medical College of Georgia at Augusta University, Augusta, GA, USA; Cardio-Oncology Program, Department of Medicine, Cardiology Division, Medical College of Georgia at Augusta University, Augusta, GA, USA; Cardio-Oncology Program, Department of Medicine, Cardiology Division, Medical College of Georgia at Augusta University, Augusta, GA, USA; Division of Hematology and Oncology, Department of Medicine, University Hospitals/Seidman Cancer Center, Case Western Reserve University School of Medicine, Cleveland, OH, USA; Faculdade Israelita de Ciências da Saúde Albert Einstein, Hospital Israelita Albert Einstein, São Paulo, SP, Brazil; Department of Medicine, Division of Cardiology, and Vascular Biology Center, Medical College of Georgia at Augusta University, Augusta, GA, USA; Cardio-Oncology Program, Department of Medicine, Cardiology Division, Medical College of Georgia at Augusta University, Augusta, GA, USA; Department of Internal Medicine, Medical College of Georgia at Augusta University, Augusta, GA, USA; Medical College of Georgia at Augusta University, Augusta, GA, USA; Medical College of Georgia at Augusta University, Augusta, GA, USA; Medical College of Georgia at Augusta University, Augusta, GA, USA; Medical College of Georgia at Augusta University, Augusta, GA, USA; Medical College of Georgia at Augusta University, Augusta, GA, USA; Medical College of Georgia at Augusta University, Augusta, GA, USA; Medical College of Georgia at Augusta University, Augusta, GA, USA; Medical College of Georgia at Augusta University, Augusta, GA, USA; Division of Hematology and Oncology, Department of Medicine, University Hospitals/Seidman Cancer Center, Case Western Reserve University School of Medicine, Cleveland, OH, USA; Cancer Population Sciences, Case Comprehensive Cancer Center, Case Western Reserve University, Cleveland, OH, USA; Division of Medical Oncology, Department of Internal Medicine, Huntsman Cancer Institute, University of Utah, Salt Lake City, UT, USA; Georgia Prevention Institute, Medical College of Georgia at Augusta University, Augusta, GA, USA; Department of Biostatistics, Epidemiology, & Informatics, Perelman School of Medicine, University of Pennsylvania, Philadelphia, PA, USA; Division of Cardiology, Department of Medicine, Perelman School of Medicine, University of Pennsylvania, Philadelphia, PA, USA; Section of Cardiovascular Medicine, Department of Medicine and Department of Radiology and Biomedical Imaging, Yale University School of Medicine, New Haven, CT, USA; Department of Medicine, Division of Cardiology, and Vascular Biology Center, Medical College of Georgia at Augusta University, Augusta, GA, USA; Cardio-Oncology Program, Department of Medicine, Cardiology Division, Medical College of Georgia at Augusta University, Augusta, GA, USA; Georgia Prevention Institute, Medical College of Georgia at Augusta University, Augusta, GA, USA; Department of Medicine, Division of Cardiology, and Vascular Biology Center, Medical College of Georgia at Augusta University, Augusta, GA, USA; Cardio-Oncology Program, Department of Medicine, Cardiology Division, Medical College of Georgia at Augusta University, Augusta, GA, USA

## Abstract

The role of social determinants of health (SDOH) in controlling hypertension (HTN) in cancer patients is unknown. We hypothesize that high SDOH scores correlate with uncontrolled HTN in hypertensive cancer patients. In our prospective study, patients completed the Protocol for Responding to & Assessing Patients’ Assets, Risks & Experiences questionnaire. After integrating home and clinic blood pressure readings, uncontrolled HTN was defined as systolic blood pressure greater than or equal to 140 mm Hg and/or diastolic blood pressure greater than or equal to 90 mm Hg. Using Cox regression, we analyzed the impact of SDOH on HTN control, adjusting for relevant factors. The study involved 318 participants (median age 66.4, median follow-up 166 days, SDOH score 6.5 ± 3.2), with stress, educational insecurity, and social isolation as prevalent adverse SDOH. High SDOH scores led to 77% increased risk of uncontrolled HTN (adjusted hazards ratio = 1.77; 95% confidence interval = 1.10 to 2.83, *P* = .018). Urban residents with high SDOH scores were at an even greater risk. Identifying SDOH and mitigating underlying factors may help control HTN, the most typical disease process treated in all cardio-oncology clinics.

Hypertension (HTN) is the most common comorbidity seen in cancer patients (1,2) and is challenging to control due to shared risk factors and metabolic effects of anticancer medications (3). Despite HTN being a statistically significant modifiable cardiovascular risk factor, achieving optimal management in cancer patients remains challenging, with only a minority achieving targeted blood pressure (BP) control (4). Modifiable lifestyle factors and social determinants of health (SDOH) are significantly associated with HTN control in the general population (5-9). It is also known that SDOHs play a crucial role in cardiovascular outcomes of patients with cancer (10,11). We hypothesized that higher SDOH scores are associated with uncontrolled HTN in cancer patients.

This Augusta University Institutional Review Board-approved prospective cohort study was conducted at a cardio-oncology clinic, enrolling adult cancer patients (≥18 years) who had at least 2 follow-up visits and had an ongoing clinical problem of HTN. The primary exposure was SDOH, assessed through the Protocol for Responding to & Assessing Patients’ Assets, Risks, & Experiences (PRAPARE) questionnaire during each participant’s first visit (12). PRAPARE, a validated tool (13-15), was organized into 6 categories for analysis: education insecurity, housing insecurity, material insecurity, transportation insecurity, social isolation, and general stress (see Supplementary Table 1, available online for details of the questionnaire). The dichotomization of the SDOH score was first identified using spline regression (Supplementary Methods, available online).

The primary outcome was uncontrolled HTN, defined as having an averaged combined home and clinic BP at or above the threshold set by the 2021 International Cardio-Oncology Society Consensus guidelines for cancer patients, specifically systolic BP (SBP) more than 140 mm Hg and diastolic BP (DBP) more than 90 mm Hg, by the last available follow-up (16). This threshold was consistent regardless of the type of cancer treatment (Supplementary Methods, Supplementary Table 6 and Figure 2, available online) (16).

The covariates gathered for each participant included age, self-reported race, sex, body mass index, diabetes mellitus, obesity, smoking status, alcohol drinking, chronic kidney disease, obstructive sleep apnea, cancer type, cancer treatment, and cancer metastasis. Additional data on lifestyle habits and comorbid conditions relevant to HTN control were also collected. The details of each covariate are presented in Supplementary Table 2 (available online).

Baseline characteristics of the cohort were summarized using median and interquartile ranges (IQR) or mean and standard deviation for continuous variables and frequencies for categorical variables. To evaluate the association between covariates and SDOH scores (high vs low), we used the χ^2^ test for categorical variables and the *t* test or Mann-Whitney *U* test for continuous variables, depending on their normality. Time-to-event was defined as the duration from enrollment to the last follow-up date for those with our outcome of uncontrolled hypertension since the study captures the time-varying nature of HTN measurements. The last follow-up date was defined as the last available clinic visit, death, or loss of follow-up date after at least 2 clinic visits. Only those without adequate follow-up (fewer than 2 clinic visits) were excluded for analysis, ensuring adequate time for monitoring and adjustment of hypertension treatment. A proportional hazards assumption testing was conducted before running the Cox proportional hazards model adjusted with covariates mentioned above, with results presented as adjusted hazard ratios (aHR) with 95% confidence intervals (CI; 2-sided *P* value of less than .05 was statistically significant). We conducted subgroup analyses, stratifying by age, sex, race, breast cancer only, cancer medication, and rurality to explore potential association variations within specific subgroups. There was a significant degree of missingness in the reported income question in the PRAPARE questionnaire (31.4%). The income data were determined to be missing at random (MAR) (Supplementary Figures 3 and 4, available online); we used multiple imputation logistic regression to generate 20 imputed datasets and after recalculating the SDOH cutoff score, and the final adjusted analysis (Supplementary Table 4, available online) was presented. The statistics were performed using Stata/MP 17.0 analytical software (Stata Corp, College Station, TX). The data were reported based on Strengthening the Reporting of Observational Studies in Epidemiology (STROBE) guidelines (17).

The initial cohort comprised 350 participants, with 318 (90.9% follow-up rate) included in the final analysis. The median age was 66 years (IQR 56-74), with 55% females, 41.5% non-Hispanic Black participants, 23.6% who had breast cancer (most common), and 81.8% who had urban areas of residence. The median follow-up duration was 166 days (IQR 67-286). The cohort’s mean SDOH score was 6.5 ± 3.2, with a high SDOH cutoff identified at greater than or equal to 5 (Supplementary Figure 1, available online). In total, 67% of patients were above this cutoff SDOH score (N = 213). Non-Hispanic Black individuals (41.5%), and urban individuals (81.8%) had statistically significant (*P* < .05) higher risk of having high SDOH scores compared with non-Hispanic White and rural participants, respectively (Table 1).

**Table 1.** Description of the cohort

		Low risk tally score (0-4)	High risk tally score (≥5)	*P*
	Total (318)	(n=105)	(n=213)	
**Demographics**				
Age (median, Q1-Q3)	67 (58-74)	67 (58-74)	66 (58-75)	.70
Older adults (≥65 years, n, %)	178 (56.0)	59 (56.2)	119 (55.9)	.96
Female (n, %)	175 (55.0)	60 (57.1)	115 (53.9)	.60
Male	143 (45.0)	45 (42.9)	98 (46.1)	
Self-reported race (n, %)				<.001
Non-Hispanic White participants	174 (54.7)	74 (70.5)	100 (46.9)	
Non-Hispanic Black participants	132 (41.5)	29 (27.6)	103 (48.4)	
Hispanic	8 (2.5)	0 (0.0)	8 (3.8)	
Others	4 (1.3)	2 (1.9)	2 (0.9)	
Rurality (n, %)				.015
Urban	260 (81.8)	78 (74.3)	182 (85.4)	
Rural	58 (18.2)	27 (25.7)	31 (14.6)	
**Lifestyle characteristics**				
Smoking status (n, %)				.35
0 (Never smoker)	153 (48.1)	50 (47.6)	103 (48.4)	
1 (Smoker)	70 (22.0)	19 (18.1)	51 (23.9)	
2 (Past history of smoking)	95 (29.9)	36 (34.3)	59 (27.7)	
Alcohol consumption (n, %)				.91
0 (Non-drinker)	91 (28.6)	29 (27.6)	62 (29.1)	
1 (Current Drinker)	120 (37.7)	39 (37.1)	81 (38.0)	
2 (Past history of drinking)	107 (33.6)	37 (35.2)	70 (32.9)	
**Comorbidities (n, %)**				
CKD	39 (12.3)	7 (6.7)	32 (15)	.033
OSA	22 (6.9)	6 (5.7)	16 (7.5)	.55
DM	97 (30.5)	36 (34.3)	61 (28.6)	.30
Obese (≥30 BMI, kg/m^2^)	138 (43.4%)	49 (46.7)	89 (41.8)	.41
**Cancer characteristics (n, %)**				
Metastatic cancer	165 (51.9%)	60 (57.1)	105 (49.3)	.17
Cancer medication	174 (54.7)	62(59.1)	112 (52.5)	.33
Breast cancer	75 (23.6%)	23 (21.9)	52 (24.4)	.62
**SDOH characteristics (n,%)**				
Housing insecurity	24 (7.5)	0 (0)	24 (11.3)	<.001
Education insecurity	197 (61.9)	44 (41.9)	153 (71.8)	<.001
Material insecurity	115 (36.2)	35 (33.3)	80 (37.6)	.46
Transportation insecurity				<.001
0 (No)	284 (89.3)	105 (100)	179 (84.0)	
1^a^	24 (7.5)	0 (0)	24 (11.3)	
2^b^	10 (3.1)	0 (0)	10 (4.7)	
Socially isolated	179 (56.3)	28 (26.7)	151 (70.9)	<.001
Stressed	247 (77.7)	65 (61.9)	182 (85.4)	<.001

a Transportation needs have hindered participants from medical appointments or getting medications. Q1-Q3 = 25th-75th percentiles; BMI = Body Mass Index; CKD = chronic kidney disease; OSA = obstructive sleep apnea; DM = diabetes mellitus; SDOH = social determinants of health.

b Transportation needs have hindered participants from medical and nonmedical appointments.

Stress from various causes (77.7%), educational insecurity (62.3%), and social isolation (56.8%) were the most common areas of adverse SDOH identified. Housing insecurity affected 7.6% of our participants with high SDOH scores. Significant differences were observed between SDOH groups (*P* < .001) in education insecurity (62.3%, with high school or less), transportation insecurity (10.6%), socially isolated (56.3%), and stressed (77.7%). Risk factor comparisons between high vs low SDOH groups are detailed in Table 1. Uncontrolled HTN was observed in 32.3% of participants. Taking cancer medication was associated with uncontrolled HTN, although not statistically significant (aHR = 1.66, 95% CI = 0.87 to 3.18, *P* = .126).

In a fully adjusted model, those with a high SDOH score showed a substantial 77% increased risk of uncontrolled HTN (aHR = 1.77, 95% CI = 1.10 to 2.83, *P* = .018) (Table 2).

**Table 2.** Cox proportional hazards model for the outcome of uncontrolled hypertension in the entire cohort, females, males, non-Hispanic Black participants, non-Hispanic White participants, older adults and non-older adult patients, rural and urban residing patients, those with breast cancer, as well as those taking cancer medications

				HR (95% CI, *P*)		
Outcome – uncontrolled HTN greater than or equal to 140/90	PRAPARE risk tally score	time at risk (person-months)	Univariable	Model 1	Model 2	Model 3
**Events/Total** ^a^						
**All population (n = 318)**						
**102/318**	0-4 (n = 105)	2025	Reference	Reference	Reference	Reference
	5+ (n = 213)		1.78 (1.13-2.81, *P = *.013)	1.75 (1.11-2.76, *P = *.017)	1.80 (1.13-2.87, *P = *.014)	1.77 (1.10-2.83, *P = *.018)
**Female (n = 175)**						
**49/175**	0-4 (n = 60)	1112	Reference	Reference	Reference	Reference
	5+ (n = 115)		1.73 (0.93-3.24, *P = *.085)	1.80 (0.96-3.38, *P = *.066)	1.78 (0.91-3.50, *P = *.092)	1.50 (0.76-2.98, *P = *.246)
**Male (n = 143)**						
**53/143**	0-4 (n = 45)	913	Reference	Reference	Reference	Reference
	5+ (n = 98)		1.72 (0.88-3.36, *P = *.114)	1.73 (0.88-3.38, *P = *.109)	1.67 (0.84-3.31, *P = *.140)	1.78 (0.89-3.55, *P = *.103)
**Non-Hispanic Black participants (n = 132)**						
**46/132**	0-4 (n = 29)	822	Reference	Reference	Reference	Reference
	5+ (n = 103)		2.25 (0.88-5.73, *P = *.089)	2.21 (0.87-5.64, *P = *.096)	2.44 (0.92-6.44, *P = *.073)	2.45 (0.92-6.52, *P = *.074)
**Non-Hispanic White participants (n = 174)**						
**51/174**	0-4 (n = 74)	1114	Reference	Reference	Reference	Reference
	5+ (n = 100)		1.57 (0.88-2.77, *P = *.124)	1.62 (0.89-2.95, *P = *.113)	1.65 (0.88-3.11, *P = *.119)	1.56 (0.82-2.96, *P = *.171)
**Older adults (n = 178)**						
**62/178**	0-4 (n = 59)	1134	Reference	Reference	Reference	Reference
	5+ (n = 119)		1.69 (0.97-2.93, *P = *.064)	1.73 (0.99-3.03, *P = *.054)	1.81 (1.01-3.23, *P = *.046)	1.77 (0.99-3.17, *P = *.054)
**Non-older adults (n = 140)**						
**40/140**	0-4 (n = 46)	891	Reference	Reference	Reference	Reference
	5+ (n = 94)		2.22 (0.97-5.06, *P = *.058)	2.22 (0.94-5.25, *P = *.070)	2.23 (0.92-5.42, *P = *.075)	1.88 (0.77-4.62, *P = *.168)
**Rural (n = 58)**						
**21/58**	0-4 (n = 27)	351	Reference	Reference	Reference	Reference
	5+ (n = 31)		1.19 (0.48-2.98, *P = *.705)	1.03 (0.40-2.63, *P = *.955)	1.10 (0.33-3.63, *P = *.876)	1.11 (0.33-3.78, *P = *.869)
**Urban (n = 260)**						
**81/260**	0-4 (n = 78)	1674	Reference	Reference	Reference	Reference
	5+ (n = 182)		2.03 (1.18-3.46, *P = *.010)	2.02 (1.18-3.46, *P = *.011)	2.05 (1.18-3.56, *P = *.011)	2.03 (1.16-3.54, *P = *.013n)
**Breast cancer (n = 75)**						
**17/75**	0-4 (n = 23)	436	Reference	Reference	Reference	Reference
	5+ (n = 52)		0.87 (0.29-2.61, *P = *.809)	0.76 (0.25-2.31, *P = *.634)	1.17 (0.32-4.31, *P = *.814)	1.07 (0.28-4.14, *P = *.924)
**Cancer medications (n = 174)**						
**54/174**	0-4 (n = 62)	1151	Reference	Reference	Reference	Reference
	5+ (n = 112)		1.75 (0.95-3.23, *P = *.074)	1.56 (0.83-2.91, *P = *.164)	1.66 (0.88-3.16, *P = *.121)	1.66 (0.87-3.18, *P = *.126)

a Based on the univariable analysis. PRAPARE = the Protocol for Responding to and Assessing Patients’ Assets, Risk, and Experiences; HR = hazards ratio; CI = confidence interval; HTN = hypertension.

Model 1: Adjusted for age and sex.

Model 2: Model 1 + diabetes mellitus, obesity, smoking status, alcohol drinking, chronic kidney disease, and obstructive sleep apnea.

Model 3: Model 2 + cancer metastasis and cancer treatment.

Among older adult participants, those with high SDOH scores had a significantly higher risk when adjusted for demographics and HTN risk factors (aHR = 1.81, 95% CI = 1.01 to 3.23), but the risk was not significant in the full model (aHR = 1.77, 95% CI = 0.99 to 3.17). Among female and non-Hispanic Black participants, those with high SDOH scores had a higher risk of uncontrolled HTN in a fully adjusted model, but this risk was not statistically significant (aHR = 1.50, 95% CI = 0.76 to 2.98 and aHR = 2.45, 95% CI = 0.92 to 6.52, respectively). Urban residents with high SDOH scores had a significantly increased risk of uncontrolled HTN (aHR = 2.03, 95% CI = 1.16 to 3.54). No significant associations were found for rural residents, males, non-Hispanic White participants, and breast cancer patients. The results of subgroup analyses are exploratory due to sample size limitations in specific subgroups.

We found no differences between participants with and without missing income data (Supplementary Table 3, available online). After imputing the missing income variable, 73.5% had high SDOH scores (≥5). In the entire cohort, older adults, and urban subgroups, the high SDOH risk for uncontrolled HTN persisted post-imputation but remained non-statistically insignificant (aHR = 1.19, 95% CI = 0.73 to 1.93, aHR = 1.70, 95% CI = 0.88 to 3.26, and aHR = 1.57, 95% CI = 0.88 to 2.78 respectively; Supplementary Table 4, available online). As part of the sensitivity analysis, we recategorized SDOH scores into low (<5), moderate (5-8), and high (≥9) risk categories. In the fully adjusted model, the aHR for the moderate and high-risk category was 1.56 (95% CI = 0.93 to 2.63) and 1.70 (95% CI = 0.99 to 2.91), respectively, indicating a clear trend toward higher risk of uncontrolled HTN with increasing SDOH scores (Supplementary Table 5, available online). The study investigated the role of adverse SDOH, measured by the PRAPARE SDOH score, in uncontrolled HTN among cancer patients undergoing HTN therapy. Stress from various causes, educational insecurity, and social isolation were the most typical areas of adverse SDOH. As hypothesized, a high SDOH score increased the risk of uncontrolled HTN by 77%. This finding was prominent in those residing in urban areas and older adults.

Our findings contribute significantly to understanding SDOHs’ role in HTN control, marking the first prospective cohort study linking SDOH burden to uncontrolled HTN in a cardio-oncology setting. Notably, the mean SDOH score of 6.5 was similar to the nationally reported average score of 7.2 by Weir et al. (12). Variables such as non-Hispanic Black participants’ race, Hispanic ethnicity, limited proficiency in English language, lower socioeconomic status, lower education levels, rural residence, and insurance status all have been previously documented as contributors to HTN control in adults (18-22). The study’s approach, focusing on composite SDOH scores rather than individual components, offers a more holistic view of social burdens, aligning with real-world scenarios and facilitating targeted interventions.

Despite the availability of health-care facilities in urban areas, significant SDOH determinants, including housing, education, transportation insecurity, social isolation, and stress, were identified. These findings echo the Reasons for Geographic And Racial Differences in Stroke Study on SDOH and racial disparities in HTN control (23-26). Social isolation and stress were statistically significant for higher SDOH scores, aligning with literature associating uncontrolled HTN with social contagion and psychological stress (27). Although the aHR indicates a trend toward higher risk in our non-Hispanic Black participants, the lack of statistical significance suggests that other factors may be crucial in influencing uncontrolled HTN. Significant disparities exist in cardiovascular outcomes and hypertension between non-Hispanic Black and non-Hispanic White participants, especially in the southeastern United States (24).

Several limitations to the presented investigation include unverified medication adherence and cross-sectional SDOH data collection. However, it is essential to note that SDOHs are known causes of medication nonadherence (28). Also, we used snapshot clinic BP measurements during the follow-up period after 2 weeks of home BP readings to determine uncontrolled hypertension. This approach may not account for the daily BP variability, potentially leading to misclassification. However, averaging multiple BP readings is the recommended method of HTN measurement by several guidelines (23, 29-31). The results of subgroup analyses were exploratory due to sample size limitations in specific subgroups. Lastly, considering the predominance of urban residents in our cohort, we acknowledge the potential for reverse causality. Our research adds to the growing evidence of the association between urban residence and the risk of uncontrolled HTN, highlighting the complex interplay between SDOH and urban living conditions (32).

In summary, our study shows that cancer patients with a high SDOH burden are at increased risk of uncontrolled HTN, even with standard medical care. This risk is especially pronounced among urban residents and older adults. Identifying adverse SDOH and mitigating underlying factors through community-based strategies and health-care policy reforms, as highlighted by Tse et al., may help control HTN, the most common disease process treated in all cardio-oncology clinics (33).

## Supplementary Material

pkae064_Supplementary_Data

## Data Availability

The data underlying this article cannot be shared due to institutional-level data that risks the privacy of individuals who participated in the study. Summary data may be available to interested researchers based on a Data Use Agreement upon reasonable request.

## Notes

**Manuscript Contributions**: **Planning of study:** AG conceived the study concept, and the study design was by PN, NS, and OMM. **Conduct of study:** PN, OMM, and NS had full access to all the data in the study and take responsibility for the integrity of the data and the accuracy of the data analysis. **Reporting of study:** PN and AG drafted the first version of the manuscript. All authors provided critical revision of the manuscript for important intellectual content. **Ethical approval information:** The Augusta University Institutional Review Boards approved this study.
