# Supplementary material for: Adverse social determinants of health elevate uncontrolled hypertension risk: a cardio-oncology prospective cohort study
Source: JNCI Cancer Spectr. 2024 Aug 8;8(5):pkae064. doi: 10.1093/jncics/pkae064 (PMC11368120; doi:10.1093/jncics/pkae064)

Title: Adverse Social Determinants of Health Elevate Uncontrolled Hypertension Risk: A Cardio-Oncology Prospective Cohort Study.

Supplementary

Content of Supplementary:

1. Supplementary methods: Spline regression, Uncontrolled Hypertension outcome:

   Description of logistic regression method used with restricted cubic spline to determine SDOH tally score cutoff.

2. Supplementary table 1: SDOH Risk Tally Score

   Details on how the SDOH Risk Tally Score was calculated using the PRAPARE questionnaire.

3. Supplementary table 3: Covariate Matrix

   A matrix showing the demographics, lifestyle characteristics, comorbidities, and cancer characteristics of the study cohort.

4. Supplementary table 4: Cox proportional hazard models using imputed PRAPARE SDHOH Risk Tally Score.

5. Supplementary table 5: Sensitivity analysis by recategorizing SDOH score as low (0-4) moderate (5-8) and high risk (>9)

6. Supplementary Table 6: Comparison of home and clinic BP measurements of entire cohort.

7. Supplementary figure 1: Cubic spline regression

8. Supplementary figure 2 and table 5: Comparison of home and clinic BP visit readings.

9. Supplementary figure 3: Missing value plot see association between missing income variable and other variables.

10. **Supplementary Figure 4**: Regression analysis to explore patterns of income variable missingness between levels of included variables

Supplementary methods:

Spline regression: The social determinants of health (SDOH) tally score cutoff was determined using logistic regression with restricted cubic splines to model its relationship with uncontrolled blood pressure[1]. This method accommodates the variable impact of each SDOH tally score increment on the OR for uncontrolled BP and assesses if the relationship is linear (each tally score point increases the OR equally) or non-linear (each tally score point increases the OR differently). The final cutoff was chosen at the score where the OR exceeded 1, considering the confidence interval.

Uncontrolled Hypertension outcome: A dual strategy was employed to ascertain BP control: Upon recruitment BP readings were first obtained by two weeks of home monitoring and thereafter BP readings were obtained by multiple routine clinical visits till the end of study period. This method ensured home and clinic BP readings were systematically integrated into the participant's clinical care. Those who failed to attend at least two follow-up clinical visit with provider were excluded from the analysis to ensure adequate time for monitoring and adjustment of hypertension treatment.. There was no significant difference in 2 weeks of home and follow up outpatient clinic BP measurement readings **(Supplementary Figure 2, Supplementary Table 6).**

Supplementary Table 1: SDOH Risk Tally Score was calculated by summing the total number of present SDOH risk responses defined by literature for an individual patient completing the full PRAPARE assessment. Higher SDOH score signifies higher burden of SDOH in participant.

**Response Categories**	**PRAPARE Tally Points by Response Category**
Ethnicity: Are you Hispanic or Latino? (Maximum of 1 tally)	
No	0
Yes	1

Race: Which race(s) are you? (Check all that apply) (Maximum of 1 tally)	
Asian	1
Native Hawaiian	1
Pacific Islander	1
Black/African American	1
American Indian/Alaskan Native	1
White	0
Other	1
Multiple Races	1
Farm Worker Status: At any point in the past 2 years, has seasonal or migrant farm work been your or your family's main source of income? (Maximum of 1 tally)	
No	0
Yes	1
Veteran Status: Have you been discharged from the armed forces of the United States? (Maximum of 1 tally)	
No	0
Yes	1
English Proficiency: What language are you most comfortable speaking? (Maximum of 1 tally)	
English	0
Language other than English	1
Generate %FPL (Maximum of 1 tally)	
100% or below	
101-150%	1
151-200%	1
200% or more	0
Unknown	1
Housing Situation: What is your housing situation today? (Maximum of 1 tally)	
I have housing	0
I do not have housing	1
Housing Stability: Are you worried about losing your housing? (Maximum of 1 tally)	
Yes (Unstable Housing)	1
No (stable housing)	0
Education: What is the highest level of school that you have finished? (Maximum of 1 tally)	
Less than high school	1
High school diploma or GED	1
More than high school degree	0
Employment: What is your current work situation? (Maximum of 1 tally)	
Unemployed and seeking work	1
Part-time work	1
Full-time work	0
Otherwise, unemployed but not seeking work	1
Insurance: What is your main insurance? (Maximum of 1 tally)	

None/uninsured	1
Medicaid	1
CHIP Medicaid	1
Other public insurance (Non-CHIP)	1
Other public insurance (CHIP)	1
Private insurance	0
Material Security: In the past year, have you or any family members you live with been unable to get any of the following when it was really needed? (Check all that apply.) (Maximum of 7 tallies)	
Food	1
Clothing	1
Utilities	1
Childcare	1

Medicine or health care	1
Phone	1
Other (enter written answer)	1
No unmet needs	0
Transportation: Has lack of transportation kept you from medical appointments, meetings, work, or from getting things needed for daily living? (Check all that apply.) (Maximum of 2 tallies)	
Yes, it has kept me from medical appointments or from getting my medications	1
Yes, it has kept me from non-medical meetings, appointments, work, or from getting things that I need	1
No transportation needs	0
Social Integration: How often do you see or talk to people that you care about and feel close to? (For example: talking to friends on the phone, visiting friends or family, going to church or club meetings) (Maximum of 1 tally)	
Less than once a week	1
1 or 2 times a week	1
3 to 5 times a week	1
More than 5 times a week	0
Stress is when someone feels tense, nervous, anxious, or can't sleep at night because their mind is troubled. How stressed are you? (Maximum of 1 tally)	
Stress: Not at all	0
A little bit	1
Somewhat	1
Quite a bit	1
Very much	1

Supplementary Table 2: Covariate Matrix

**Demographics**	**Definition**
Age	At the time of presentation the cardio-oncology clinic for visit 1.
Rurality	Defined using the 2013 National Center of Health Statistics Urban- Rural classification system[1]. Using the patient's address, those living in non-metropolitan counties with a population of <50,000 were defined as rural patients.
**Lifestyle characteristics**	
Smoking Status	0- Never smoker<br>1- Active smoker<br>2- Past history of smoking
Alcohol Consumption	0- Never history of alcohol consumption<br>1- Active drinker<br>2- Past history of alcohol consumption
**Comorbidities**	
Chronic Kidney Disease	0- No diagnosis of chronic kidney disease<br>1- Clinically diagnosed chronic kidney disease
Obstructive Sleep Apnea	0- No diagnosis of obstructive sleep apnea<br>1- Clinically diagnosed obstructive sleep apnea
Diabetes Mellitus	0- No diagnosis of Diabetes mellitus<br>1- Clinical diagnosis of Diabetes mellitus
Obese (≥30)	0- BMI<30<br>1- BMI ≥30
**Cancer characteristics**	
Metastatic cancer	0- No Documented metastatic cancer.<br>1- Documented metastatic cancer.
Breast cancer	0- No diagnosed breast cancer.<br>1- Diagnosed breast cancer.
Cancer Medications	Our assessment of cancer medications, as detailed in Supplementary Table 2, was guided by the American Heart Association's statement on their impact on blood pressure[2]. Patients' medication status during the study was dichotomously recorded: '1' for current medications and '0' for "not on current treatment

Drug Class	Examples
Tyrosine Kinase Inhibitors	Imatinib
Vascular endothelial growth factor inhibitors	Bevacizumab, Norafenib, Sunitinib, Nilotinib,<br><br>Pazopanib, Dasatinib.

Anti-HER2	Trastuzumab, Pertuzumab, Lapatinib, Neratinib.
Aromatase inhibitors	Anastrozole, Letrozole.
Androgen Deprivation Therapy	Leuprolide, Abiraterone, Enzalutamide.
Taxanes	Paciltaxel, Docetaxel.
Cyclophosphamides	Cyclophosphamide, Busulfan, Ifosfamide.
Calcineurin inhibitors	Tacrolimus, Cyclosporin.
Platinum based drugs	Cisplatin, Carboplatin and Oxaliplatin.
Steroids	

1. Ingram DD, Franco SJ. NCHS urban-rural classification scheme for counties. *Vital Health Stat 2*. 2012 Jan;(154):1-65.
2. Cohen JB, Brown NJ, Brown SA, Dent S, van Dorst DCH, Herrmann SM, Lang NN, Oudit GY, Touyz RM; American Heart Association Council on Hypertension; Council on Arteriosclerosis, Thrombosis and Vascular Biology; and Council on the Kidney in Cardiovascular Disease. Cancer Therapy-Related Hypertension: A Scientific Statement From the American Heart Association. Hypertension. 2023 Mar;80(3):e46-e57. doi: 10.1161/HYP.0000000000000224. Epub 2023 Jan 9. PMID: 36621810; PMCID: PMC10602651.

Supplementary table 3: Comparison between characteristics of those participants with missing income variable in PRAPARE questionnaire Vs those without any missing data.

	**Not missing data (n=215)**	**Missing data (n=103)**	**p-value**
**Demographics**			
Age (mean, SD)	64.1 (13.0)	65.4 (14.2)	0.45
Older Adults (≥65 years, n)	125	53	0.88
Female (n, %)	119 (55.3)	56 (44.4)	0.96
Male	96 (44.6)	47 (45.6)	
Race (n, %)			0.91
Non-Hispanic White Participants (n, %)	120 (55.8)	54 (52.4)	
Non-Hispanic Black Participants (n, %)	87 (40.5)	45 (43.7)	
Hispanic (n, %)	5 (2.3)	3 (2.9)	
Others (n, %)	3 (1.3)	1 (1)	
Rurality (n, %)			0.74
Urban	187 (87)	73 (70.9)	
Rural	38 (17.7)	20 (19.4)	
**Lifestyle characteristics (n, %)**			
Smoking Status			0.70
0 (Never Smoker)	107 (49.8)	46 (44.7)	

1 (Smoker)	45 (20.9)	25 (24.3)	
2 (Past History of Smoking)	64 (29.8)	31(30.1)	
Alcohol Consumption			0.57
0 (Non-Drinker)	66 (30.7)	25 (24.3)	
1 (Current Drinker)	78 (36.3)	42 (40.8)	
2 (Past history of drinking)	72 (33.5)	35 (34)	
**Co-morbidities (n, %)**			
CKD	30 (13.9)	9 (8.7)	0.25
OSA	14 (6.5)	8 (7.8)	0.86
DM	67 (31.2)	30 (29.1)	0.81
BMI (mean)	29.6	29.3	0.81
**Cancer characteristics (n, %)**			
Metastatic cancer	112 (52.1)	53 (51.5)	0.95
Cancer medication	43 (20)	26 (25.2)	0.36
Breast cancer	55 (25.6)	20 (19.4)	0.68
**SDOH characteristics (n, %)**			
Housing insecurity	16 (7.4)	8 (7.8)	1.0
Education insecurity	133 (61.9)	64 (62.1)	0.79
Material insecurity	77 35.8)	38 (36.9)	0.99
Transportation insecurity			0.25
0 (No)	190 (88.4)	94 (91.3)	
1[a]	14 (6.5)	10 (9.7)	
2[b]	9 (4.2)	1 (1)	

Socially isolated	126 (58.6)	53 (51.5)	0.18
Stressed	171 (79.5)	76 (73.8)	0.09

Supplementary table 4: Cox proportional hazards model for the outcome of uncontrolled hypertension in the sub-groups after imputing for missing variable in PRAPARE SDOH Risk Tally score.

Abbreviations: HR, hazards ratio; CI, confidence interval

Model 1: Adjusted for Age, and sex..

Model 2: Model 1 + diabetes mellitus, obesity, smoking status, alcohol drinking, chronic kidney disease, and obstructive sleep apnea.

Model 3: Model 2 + Cancer metastasis, cancer treatment

	**Model 1 (HR, 95% CI)**	**Model 2 (HR, 95% CI)**	**Model 3 (HR, 95% CI)**
**All population (n=318)**	1.28 (0.79- 2.08)	1.29 (0.80-2.09)	1.19 (0.73-1.93)
**Older Adults (n= 178)**	1.80 (0.96-3.37)	1.81 (0.95-3.44)	1.70 (0.88-3.26)
**Urban (n= 260)**	1.69 (0.95-3.02)	1.71 (0.96-3.06)	1.57 (0.88-2.78)

Supplementary table 5: Sensitivity analysis by recategorizing SDOH score as low (0-4) moderate (5-8) and high risk (>9)

				HR (95%CI, P-value)			
**Outcome**	**PRAPARE Risk Tally Score**	**Events/Total***	**Time at Risk (person-months)**	**Univariable**	**Model 1**	**Model 2**	**Model 3**
**All Population (n=318)**							
**Uncontrolled HTN >= 140/90**	0-4 (n=105)	102/318	2,025	Reference	Reference	Reference	Reference
	5-8 (n=126)			**1.69 (1.03-2.77, p=0.037)**	**1.66 (1.01-2.72, p=0.047)**	**1.71 (1.02-2.85, p=0.042)**	1.56 (0.93-2.63, p=0.092)
	9+ (n=87)			**1.92 (1.14-3.25, P=0.015)**	**1.88 (1.11-3.18, P=0.019)**	**1.92 (1.13-3.27, P=0.017)**	1.70 (0.99-2.91, P=0.054)
**Older Adults (n=178)**							
**Uncontrolled HTN >= 140/90**	0-4 (n=59)	62/178	1,134	Reference	Reference	Reference	Reference
	5-8 (n=69)			1.69 (0.91-3.11, P=0.095)	1.74 (0.93-3.26, P=0.081)	1.75 (0.91-3.38, P=0.096)	1.63 (0.84-3.17, P=0.149)
	9+ (n=50)			1.68 (0.88-3.22, P=0.115)	1.72 (0.90-3.29, P=0.104)	1.87 (0.95-3.66, P=0.068)	1.69 (0.86-3.33, P=0.126)
**Non- Older Adults (n=140)**							
**Uncontrolled HTN >= 140/90**	0-4 (n=46)	40/140	891	Reference	Reference	Reference	Reference
	5-8 (n=57)			2.03 (0.85-4.87, p=0.111)	2.09 (0.84-5.21, p=0.113)	2.13 (0.83-5.45, p=0.116)	1.72 (0.66-4.49, p=0.269)
	9+ (n=37)			**2.56 (1.01-6.47, P=0.047)**	2.42 (0.93-6.32, P=0.071)	2.42 (0.88-6.65, P=0.087)	1.76 (0.64-4.85, P=0.276)
**Rural (n=58)**							
**Uncontrolled HTN >= 140/90**	0-4 (n=27)	21/58	351	Reference	Reference	Reference	Reference
	5-8 (n=21)			1.20 (0.45-3.25, p=0.715)	1.06 (0.39-2.92, p=0.905)	1.37 (0.35-5.32, p=0.652)	1.24 (0.33-4.69, p=0.747)

	9+ (n=10)			1.17 (0.34-4.01, P=0.801)	0.95 (0.27-3.40, P=0.938)	0.81 (0.17-3.86, P=0.788)	0.74 (0.16-3.40, P=0.698)
			**Non-Rural (n=260)**				
**Uncontrolled HTN >= 140/90**	0-4 (n=78)	81/260	1,674	Reference	Reference	Reference	Reference
	5-8 (n=105)			**1.88 (1.05-3.36, p=0.033)**	**1.87 (1.04-3.35, p=0.037)**	**1.89 (1.04-3.44, p=0.038)**	1.72 (0.93-3.17, p=0.083)
	9+ (n=77)			**2.24 (1.22-4.10, P=0.009)**	**2.23 (1.22-4.09, P=0.009)**	**2.27 (1.23-4.21, P=0.009)**	**1.96 (1.04-3.68, P=0.036)**

Supplementary Table 6: Comparison of home and clinic BP measurements of entire cohort.

N=318	Home BP	Clinic BP	P value
SBP (Mean, Q1-Q3)	134.9 (120-140)	131.3 (120.5-142.5)	0.36
DBP (Mean, Q1-Q3)	72.01 (63.95-79.93)	72.03 (64.46-79.81)	0.87

Supplementary Figure 1: Cubic spline regression to find the cutoff for the social determinants of health tally score for Cox models.

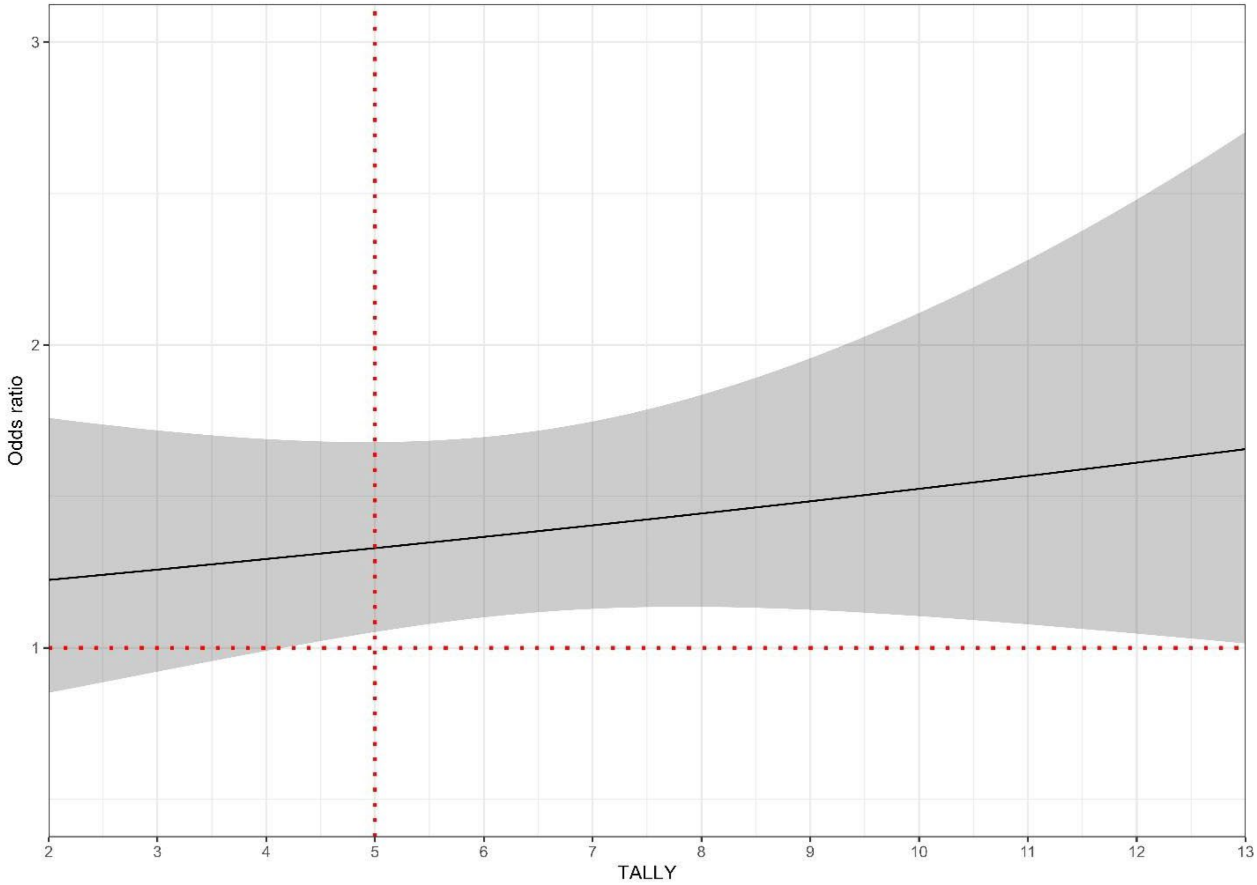

References:

1. Stabellini N, Dmukauskas M, Bittencourt MS, Cullen J, Barda AJ, Moore JX, Dent S, Abdel-Qadir H, Kawatkar AA, Pandey A, Shanahan J, Barnholtz-Sloan JS, Waite KA, Montero AJ, Guha A. Social Determinants of Health and Racial Disparities in Cardiac Events in Breast Cancer. *J Natl Compr Canc Netw.* 2023 Jul;21(7):705- 714.e17.

Supplementary Figure 2: Comparison of averaged home BP readings and averaged follow up clinic visit BP readings of first 20 pilot cohort participants. There was no statistical difference observed in home and clinic BP readings.

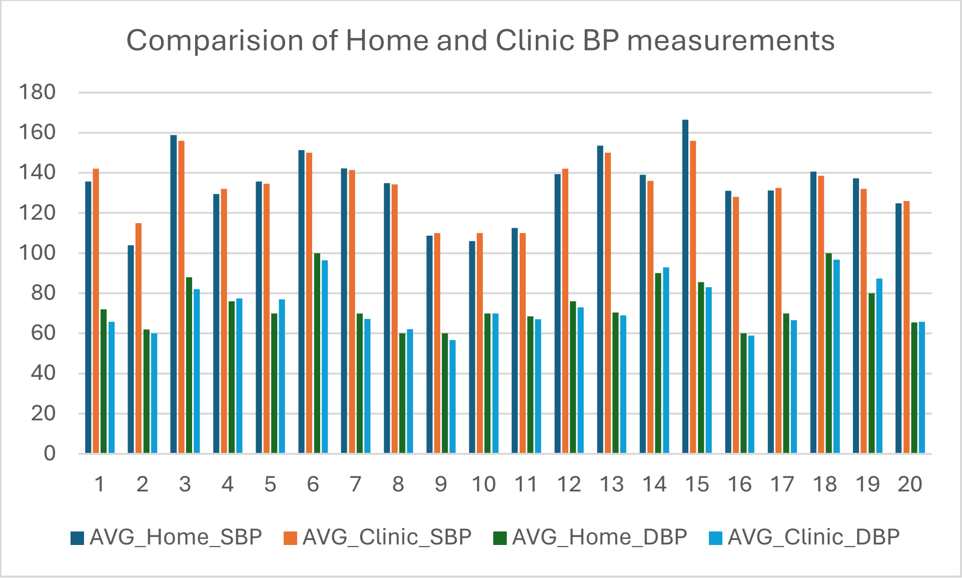

Supplementary Figure 3- Missing value plots to see association between missingness and observation[1]. No repeated pattern of missingness between patients that don't have the income information was noted.

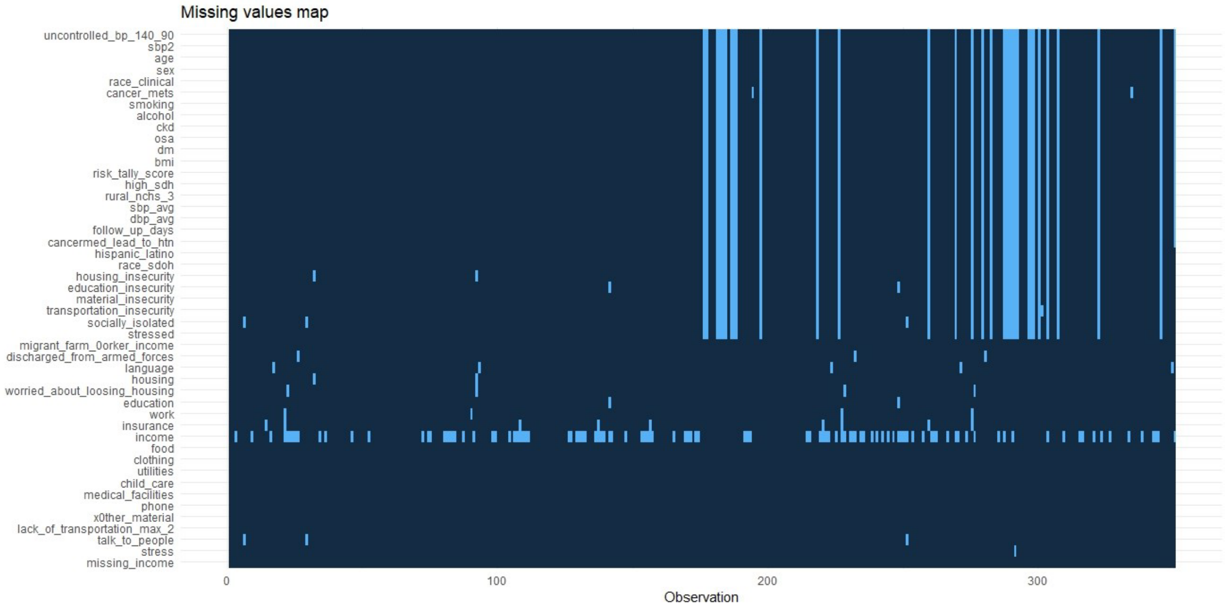

1. Sterne JA, White IR, Carlin JB, Spratt M, Royston P, Kenward MG, Wood AM, Carpenter JR. Multiple imputation for missing data in epidemiological and clinical research: potential and pitfalls. BMJ. 2009 Jun 29;338:b2393. doi: 10.1136/bmj.b2393. PMID: 19564179; PMCID: PMC2714692.

Supplementary Figure 4: Regression analysis to explore patterns of income variable missingness between levels of included variables.

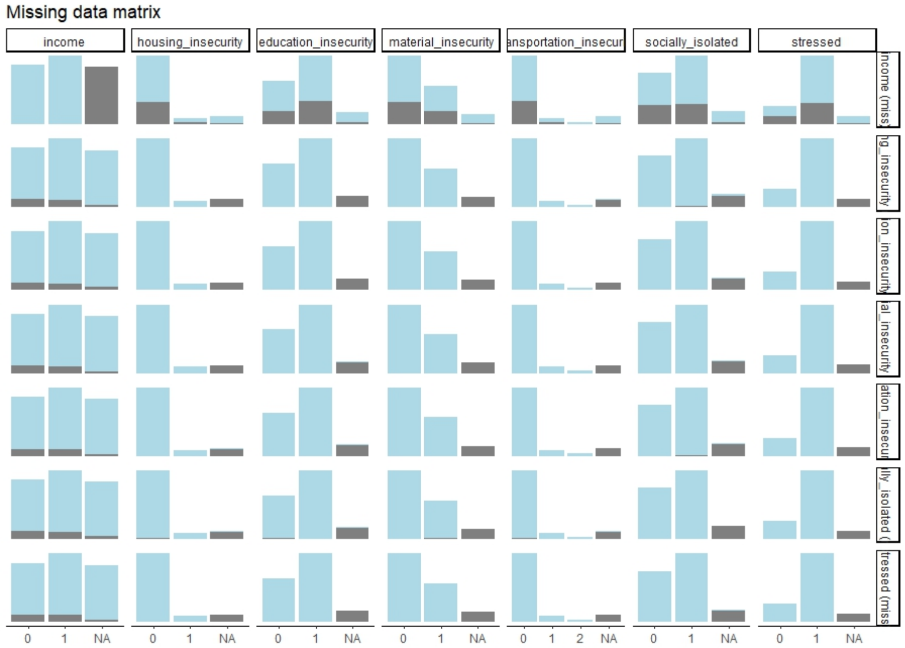

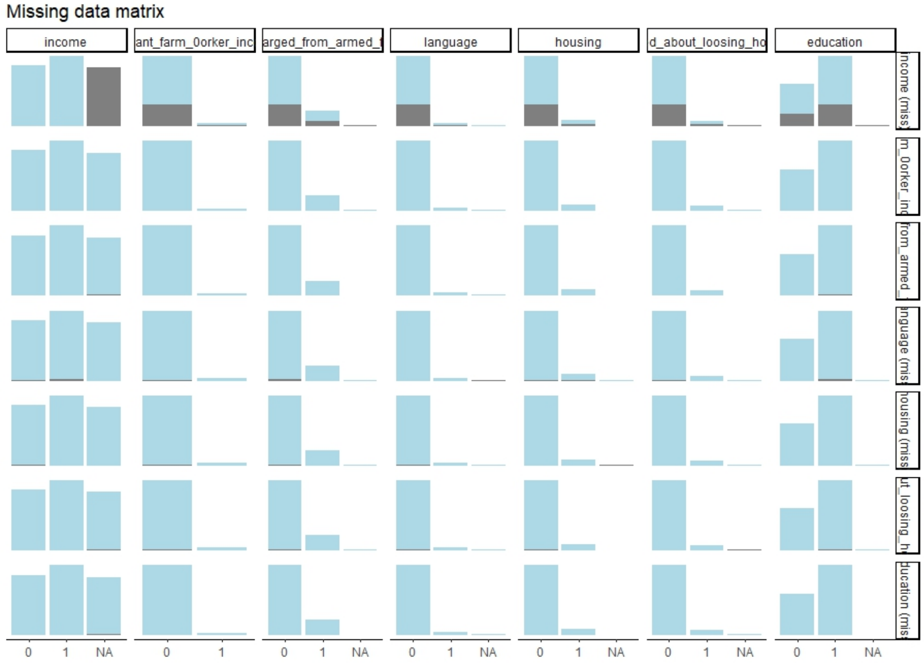

Missing data matrix

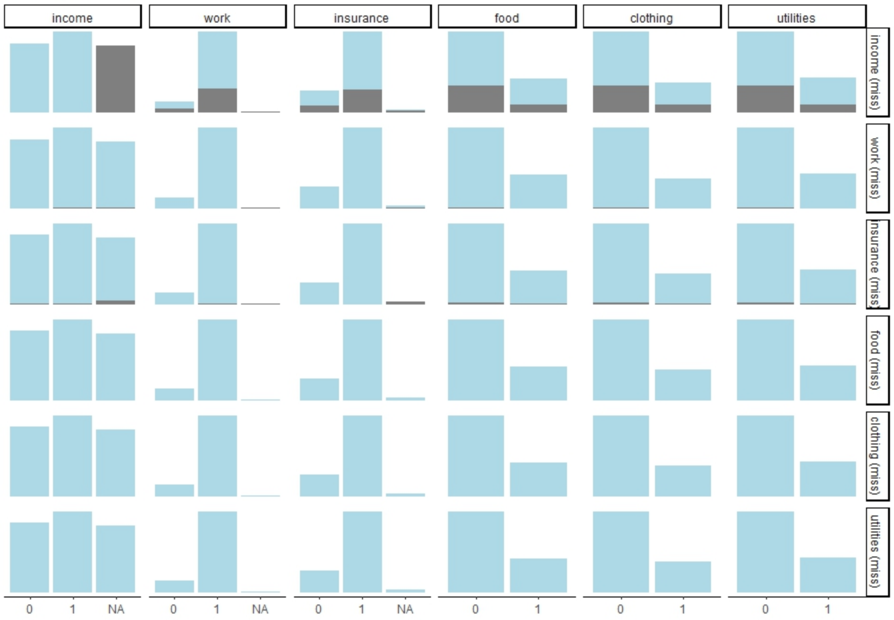

Missing data matrix

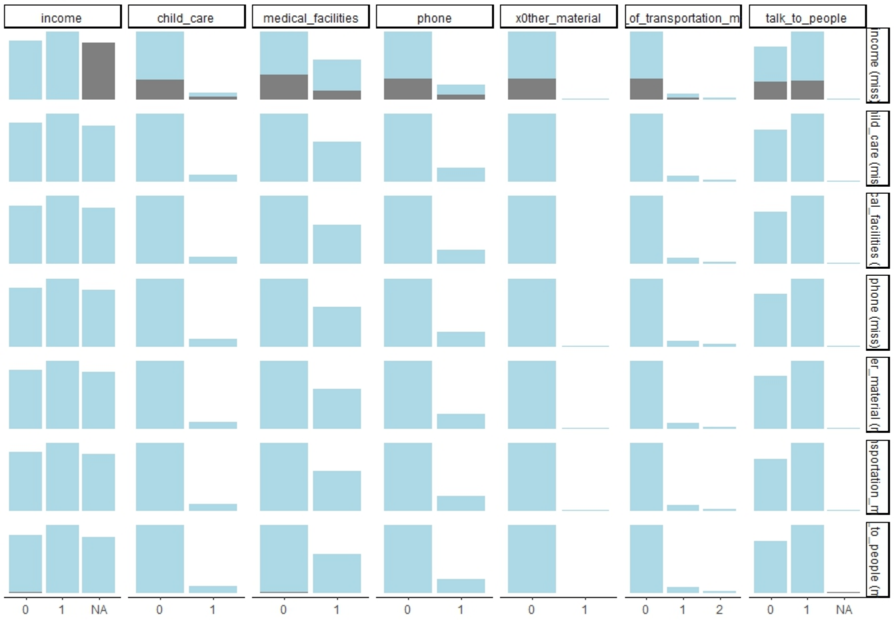

Supplement: pkae064_Supplementary_Data [file pkae064_supplementary_data.pdf]
